# Menin Inhibition in Acute Myeloid MLL Rearranged Leukemias: A New Target for Precision Care

**DOI:** 10.3390/cancers18040637

**Published:** 2026-02-15

**Authors:** Caterina Alati, Matteo Molica, Martina Pitea, Violetta Marafioti, Gaetana Porto, Giorgia Policastro, Erica Bilardi, Giovanna Utano, Laura Giordano, Annalisa Sgarlata, Ilaria Maria Delfino, Aurora Idato, Giulia Santoro, Marco Rossi, Massimo Martino

**Affiliations:** 1 Hematology and Stem Cell Transplantation and Cellular Therapies Unit (CTMO), Department of Hemato-Oncology and Radiotherapy, Grande Ospedale Metropolitano “Bianchi-Melacrino-Morelli”, 89133 Reggio Calabria, Italyerica.bilardi@ospedalerc.it (E.B.); massimo.martino@ospedalerc.it (M.M.); 2 Division of Hematology-Oncology, Azienda Universitaria Ospedaliera Renato Dulbecco, 88100 Catanzaro, Italy; 3 Hematology, Stem Cell Transplantation, Fondazione Policlinico Universitario Campus Bio Medico, 00128 Rome, Italy

**Keywords:** menin inhibitors, KMT2A rearrangements, MLL rearrangements, NPM1 mutations, acute myeloid leukemia, revumenib, precision medicine, targeted therapy

## Abstract

Menin inhibitors are the first targeted therapies for KMT2A-rearranged and NPM1-mutated acute leukemias, addressing a significant unmet need in these high-risk subtypes. Revumenib received approval in 2024–2025 for relapsed or refractory KMT2A-rearranged acute leukemia and NPM1-mutated AML. The AUG-MENT-101 trial reported a 23% composite complete remission rate in heavily pretreated patients, with 61% of responders achieving MRD negativity. Several menin inhibitors, including ziftomenib, bleximenib, and enzomenib, are in clinical development. They demonstrate similar efficacy, but their safety profiles differ, especially regarding QTc prolongation and coverage of resistance mutations. Combination therapies with azacitidine and venetoclax or intensive chemotherapy have achieved high response rates in newly diagnosed patients, supporting their potential use in frontline treatment. Acquired resistance, often due to MEN1 mutations at the drug-binding interface, occurs in about 40% of cases. Distinct resistance patterns among menin inhibitors suggest the possibility of sequential therapy. Approximately 30–40% of responders in relapsed or refractory trials proceeded to allogeneic transplantation, which remains a key pathway to potential cure. This review examines the molecular mechanisms of the menin-KMT2A interaction, and summarizes clinical trial data on the efficacy and safety of menin inhibitors as monotherapy and in combination.

## Simple Summary

Menin inhibitors are new targeted drugs for two high-risk types of acute leukemia (KMT2A-rearranged and NPM1-mutated). Revumenib was approved in 2024–2025. In heavily pretreated patients, about 23% achieved complete remission, with most of those becoming MRD-negative. Ziftomenib, bleximenib, and enzomenib show similar effectiveness but differ in side effects, particularly heart rhythm problems (QTc prolongation varies among drugs). When combined with other drugs like azacitidine/venetoclax or chemotherapy, response rates are much higher in newly diagnosed patients, suggesting these could work as initial treatment. About 40% of patients develop resistance, usually through MEN1 gene mutations. Different menin inhibitors have different resistance patterns, so switching drugs might help. About 30–40% of responders went on to stem cell transplant, which remains the best chance for cure. Menin inhibitors are the first targeted therapies for these aggressive leukemias, showing promise both alone and in combinations, though resistance remains a challenge.

## 1. Introduction

Acute myeloid leukemia (AML) is a challenging hematologic malignancy with high relapse rates and limited treatment options, especially for genetically defined subgroups such as NPM1-mutant (mNPM1) [1] and KMT2A-rearranged (KMT2Ar) AML [2]. NPM1-mutated AML accounts for approximately 30% of newly diagnosed AML cases worldwide, translating to roughly 20,000 new cases annually based on global AML incidence estimates [3,4,5,6,7]. NPM1 mutations alone are considered a favorable risk [3]. However, after relapse, mNPM1 AML is reclassified as unfavorable because of a high risk of resistance to standard therapies [3,4]. KMT2A rearrangements are frequently observed in therapy-related AML and infant leukemias. The estimated annual global incidence of acute leukemias with KMT2A rearrangements is approximately 7000 cases, representing 4% to 10% of all AML cases and up to 80% of infant acute lymphoblastic leukemia [3,4,5,6,7]. KMT2Ar is most common in therapy-related AML or AML following prior radiation or chemotherapy. De novo KMT2Ar acute lymphoblastic leukemia (ALL) occurs in 10% to 15% of cases. Adults with KMT2Ar disease have a poor prognosis, with a 5-year overall survival (OS) rate below 25% [8,9]. Although intensive induction chemotherapy can induce remission, these patients often relapse quickly, even after allogeneic stem cell transplant (allo-SCT), and standard therapies are rarely effective after relapse.

NPM1-mutated AML represents a distinct molecular entity that shares critical pathogenic dependencies with KMT2A-rearranged disease (1,3,4). NPM1 (nucleophosmin) normally shuttles between the nucleus and cytoplasm, where it functions in ribosome biogenesis, centrosome duplication, and regulation of the ARF-p53 pathway. The most common NPM1 mutations (type A: c.863_864insTCTG) occur in exon 12 and result in a frameshift that disrupts nuclear localization signals while creating a novel nuclear export signal. This causes aberrant cytoplasmic accumulation of mutant NPM1, which leads to several oncogenic consequences: dysregulation of HOX gene expression through mechanisms that require intact menin-KMT2A interaction; sequestration of wild-type NPM1 (heterodimer formation), causing haploinsufficiency; disruption of ARF tumor suppressor activity.

Critically, despite distinct genetic lesions, NPM1-mutated and KMT2A-rearranged AML converge on a shared molecular dependency: both require the menin-KMT2A interaction to maintain aberrant HOX gene expression, particularly HOXA9 and MEIS1. This convergence provides the molecular rationale for extending menin inhibitor therapy from KMT2A-rearranged to NPM1-mutated disease.

**Figure 1:**
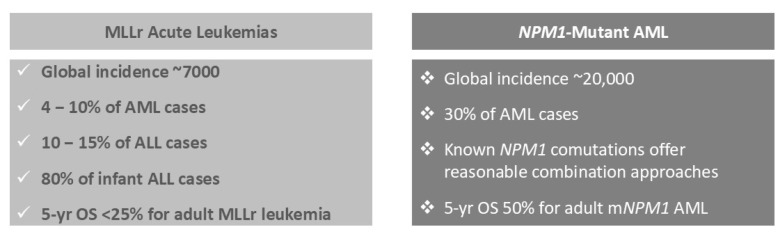
Incidence of MLLr and NPM1-Mutant Acute Leukemia.

Menin is a transcriptional adaptor protein that regulates gene expression by binding to KMT2A (formerly MLL1), leading to abnormal HOX gene expression characteristics of both KMT2Ar and mNPM1 AML [10,11,12,13] (Figure 1). Menin inhibitors disrupt this interaction, suppress oncogenic transcription, and promote differentiation of leukemic blasts into functional myeloid cells. Identifying menin as a key oncogenic cofactor in KMT2A-rearranged leukemias has led to the development of new therapeutic strategies [13,14,15,16,17]. This dependency has driven the development of small-molecule menin inhibitors that block the menin-KMT2A interaction. In November 2024, revumenib became the first FDA-approved therapy in this class for relapsed (R) or refractory (R) AL with KMT2A translocations [18,19]. In October 2025, its approval was extended to NPM1-mutated AML, potentially benefiting 40% of AML patients with these targetable mutations [20].

This review evaluates inhibition of menin activity as a precision therapy, integrating molecular mechanisms with clinical evidence. It also discusses key challenges, including resistance and optimal treatment sequencing.

## 2. Molecular Mechanisms of Mll-Menin Interaction

### 2.1. Normal KMT2A Function and Regulation

KMT2A encodes a histone H3 lysine 4 (H3K4) methyltransferase that functions in a chromatin-modifying complex of over 30 proteins [21]. In normal hematopoiesis, wild-type KMT2A regulates HOX gene expression, especially HOXA9 and its co-factor MEIS1, which are critical for hematopoietic stem cell self-renewal and differentiation [22]. The interaction between KMT2A and menin is necessary for the complex to localize to specific genomic loci and maintain proper HOX gene expression during development and hematopoietic maturation.

### 2.2. Pathogenic Role of KMT2A Rearrangements

**Figure 2:**
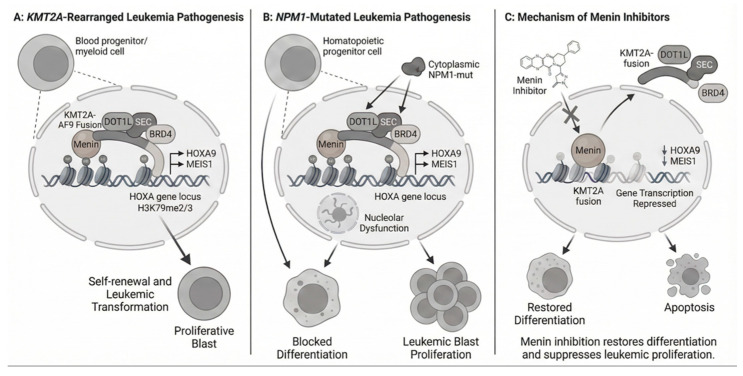
Pathogenesis of *KMT2A*-Rearranged and *NPM1*-Mutant Acute Leukemias and Mechanism of Menin Inhibitors. Panel (**A**) KMT2A-Rearranged, Panel (**B**) NPM1-Mutated, Panel (**C**) Menin Inhibitor Mechanism.

Chromosomal rearrangements at the KMT2A locus (11q23.3) produce fusion proteins that join the N-terminal portion of KMT2A to over 120 partner proteins [23] (Figure 2). Despite the diversity of partners, these rearrangements share a common pathogenic mechanism. The fusion proteins retain the menin-binding domain in the N-terminal KMT2A segment but lack the C-terminal SET domain needed for H3K4 methylation. This menin-binding domain enables the fusion proteins to associate with chromatin and drive persistent overexpression of HOXA9, MEIS1, and other downstream targets, leading to differentiation arrest and leukemic transformation.

### 2.3. The Menin-KMT2A Protein–Protein Interaction

Structural studies have defined the menin-KMT2A interaction interface [24]. KMT2A binds menin with high affinity through residues 5–43, which interact with a central binding pocket on menin formed by M327, G331, T349, and adjacent amino acids [25]. This interaction is essential for recruiting KMT2A and its fusion proteins to chromatin at HOX gene loci and for maintaining active transcription through histone modification and chromatin remodeling.

### 2.4. Mechanism of Action of Menin Inhibitors

Menin inhibitors are small molecules that competitively bind the menin pocket, blocking its interaction with KMT2A [24], thus preventing menin association with KMT2A fusion proteins and displacing them from chromatin. As a result, HOXA9, MEIS1, and other menin-KMT2A target genes are rapidly downregulated. This relieves the differentiation block, promotes myeloid maturation, and induces apoptosis in leukemic cells [26]. Menin inhibitors work across KMT2A fusion partners, providing a unified therapeutic approach for various KMT2A rearrangements [27].

### 2.5. Convergent Pathogenic Pathways: Extension to NPM1-Mutated AML

NPM1 mutations, found in approximately 30% of adult AML cases, define a distinct molecular subtype that nevertheless shares critical dependencies with KMT2A-rearranged disease [28]. The pathogenic mechanism of NPM1 mutations involves multiple interconnected pathways that converge on dysregulated HOX gene expression. The primary mechanism is cytoplasmic dislocation: frameshift mutations in exon 12 of NPM1 disrupt nuclear localization while creating a nuclear export signal, causing aberrant cytoplasmic accumulation of the mutant protein. This dislocation has pleiotropic effects on nuclear architecture and gene regulation. Moreover, despite the absence of KMT2A fusion proteins, NPM1-mutated cells exhibit HOX gene expression patterns remarkably like KMT2A-rearranged leukemia. Multiple mechanisms contribute to this convergence: (1) Loss of nuclear NPM1 disrupts normal chromatin organization, favoring permissive chromatin states at HOX loci; (2) Cytoplasmic mutant NPM1 sequesters nuclear import factors, indirectly affecting transcription factor localization; (3) Changes in chromatin accessibility enhance recruitment of wild-type KMT2A-menin complexes to HOX gene promoters and enhancers.

Another mechanism is the epigenetic reprogramming (29). NPM1 mutations alter the epigenetic landscape through interactions with histone modifiers and chromatin remodelers. This creates a cellular context where HOX gene activation becomes dependent on menin-KMT2A function, even in the absence of KMT2A rearrangements.

Finally, gene expression profiling reveals that NPM1-mutated and KMT2A-rearranged AML cluster together based on HOX/MEIS1 expression signatures, despite different initiating genetic lesions. Both subtypes exhibit high HOXA9 and MEIS1 expression, dependency on menin for maintaining these expression patterns, and sensitivity to menin inhibition-induced differentiation.

This molecular convergence on the menin-KMT2A-HOX axis provides the mechanistic rationale for extending menin inhibitor therapy beyond KMT2A rearrangements to include NPM1-mutated AML. The shared dependency explains why menin inhibitors achieve comparable response rates (~23–25% CCR) in both populations and why both exhibit similar resistance mechanisms (MEN1 mutations). Recognition of these convergent pathways has expanded the potential target population from ~7000 (KMT2Ar) to ~27,000 annual cases worldwide (KMT2Ar + NPM1m), representing approximately 40% of all AML patients with actionable molecular targets for menin inhibition [28].

## 3. Clinical Trial Data and Outcomes

### 3.1. Revumenib (SNDX-5613): The AUGMENT-101 Trial—KMT2A-Rearranged Cohort

The AUGMENT-101 trial (NCT04065399) was pivotal in supporting revumenib regulatory approval [29]. This multicenter, open-label phase 1/2 trial enrolled patients with relapsed/ refractory acute leukemia (R/R AL) and either KMT2A rearrangements or NPM1 mutations. In the KMT2A-rearranged cohort, 104 heavily pretreated adults and children received revumenib at 163 mg twice daily or a weight-adjusted dose for those under 40 kg.

Composite Complete Remission (CCR) is a broader measure than standard Complete Remission (CR). It includes patients who achieve CR (normal counts) and those in CR with incomplete count recovery (CRi), where blood counts have not fully normalized, but leukemia cells are absent [30]. The AUGMENT-101 trial reported an overall response rate (ORR) of 64% and a CCR rate of 23% (21% CR + CRi, 95% CI: 13.8–30.3%). Among patients achieving CR or CRi, the median response duration was 6.4 months. Sixty-one percent of these responders achieved minimal residual disease (MRD) negativity, and 34% proceeded to allo-SCT. The median time to CR or CRi was 1.9 months, supporting timely transition to potentially curative therapy. Based on these results, the FDA approved revumenib for R/R AL with KMT2A translocations [19].

### 3.2. Revumenib (SNDX-5613): The AUGMENT-101 Trial—NPM1-Mutated Cohort

The phase 2 of AUGMENT-101 enrolled 84 patients with NPM1-mutated R/R AML, with a median age of 63 years. Seventy-five percent had received prior venetoclax, and 35.9% had at least three prior lines of therapy. In the efficacy-evaluable group of 64 adults, the CR + CRi rate was 23.4% (*p* = 0.0014), meeting the primary endpoint. The ORR was 46.9%, and the median CCR duration was 4.7 months. Among 30 responders, 16.7% proceeded to allo-SCT, and three resumed revumenib after transplant. These results led to FDA approval for the NPM1-mutated indication in October 2025, making revumenib the first and only FDA-approved therapy for both KMT2A-rearranged and NPM1-mutated R/R AL [20].

### 3.3. AUGMENT-101: Safety Results from Phase II R/R—mNPM1 AML Cohort

Revumenib can cause QT prolongation (30). Grade 3 QT prolongation occurred in 19% of patients, and 2% experienced grade 4 events. There is a risk of differentiation syndrome (DS), with grade 3 events in 11% and grade 4 events in 2% of patients. DS is reported with all menin inhibitors and requires close monitoring. If DS is suspected, steroids such as dexamethasone 10 mg twice daily should be given promptly, as DS can become severe [29].

### 3.4. Ziftomenib (KO-539): The KOMET-001 Trial

The KOMET-001 phase I/II trial evaluated ziftomenib in patients with R/R AML [31], including multiple substudies (NCT04067336). The initial dose-escalation phase assessed safety and tolerability at doses from 50 mg to 1000 mg daily. Phase Ib included two monotherapy validation cohorts in adults with mNPM1- and KMT2Ar-R/R AML. Phase II evaluated ziftomenib at 600 mg daily in mNPM1 R/R AML. Safety and tolerability were the primary endpoints, and 600 mg was established as the recommended dose. On-target DS was identified as a potentially life-threatening complication. This led investigators to halt monotherapy in KMT2Ar disease and continue with mNPM1 R/R AML only. About one-third of newly diagnosed AML patients carry NPM1 mutations, and about half undergo relapse, requiring salvage therapy. A pooled analysis of phase Ib/II included 112 heavily pretreated patients (median age 69, range 22–86), with a median of two prior therapies (range 1–7). Approximately 25% had prior allo-SCT, 60% had prior venetoclax, and 53% had a concomitant FLT3 mutation [31].

### 3.5. KOMET-001: R/R mNPM1 AML Response

The pooled phase Ib and II cohorts showed that ziftomenib has clinical efficacy in R/R mNPM1 AML. In these heavily pretreated patients, the CCR rate was 25% (18% CR, 7% CRi), and the median DoR was 5.1 months for those achieving CRc. Sixty-five percent of CRc responders were MRD-negative by central molecular testing. Interestingly, FLT3 or IDH1/2 mAML patients as well as those with prior HSCT or venetoclax, showed an ORR similar to the overall population. These data contrast with revumenib studies, where prior venetoclax appeared to confer resistance. The phase II cohort used CCR as the primary endpoint, with a historical control CCR of 12% [32]. The observed CCR rates of 25% overall and 23% in phase II were statistically superior to the historical control [31].

### 3.6. KOMET-001: OS in Patients with R/R mNPM1 AML

In the transplant cohort, the median OS was 6.1 months. Responders had a median OS of 16.4 months, compared to 3.5 months for nonresponders, consistent with historical data. At data cutoff, 24 patients remained alive, and 9 of 92 in the phase II cohort were still on treatment [31].

There are some distinctions between the adverse events (AEs) experienced with ziftomenib and revumenib. About two-thirds of patients experienced a ziftomenib-related AE, but there was no evidence of clinically significant QTc prolongation in this trial. Three patients did experience QTc prolongation, but all had other factors that may have contributed, including other medications, electrolyte abnormalities, and a previous diagnosis of atrial fibrillation. Grade 3 or higher anemia and neutropenia were reported in about 5% of patients. DS was reported in 24% of patients, but only 13% had grade 3 events, and none had grade 4 or 5 events. Treatment-related AEs with ziftomenib led to discontinuation in 3% of patients. Overall, ziftomenib monotherapy appeared well tolerated.

Ziftomenib received orphan drug designation from the European Medicines Agency and breakthrough therapy designation from the FDA for NPM1-mutated AML [33].

### 3.7. cAMeLot-1: Phase I Trial of Bleximenib Monotherapy in R/R Acute Leukemia with KMT2Ar and mNPM1

Bleximenib is one of the most widely used menin inhibitors outside the US. It has demonstrated clinical efficacy in patients with KMT2Ar and mNPM1 AML and has shown promising activity against inhibitor-resistant mutations in preclinical studies [34]. Clinically, bleximenib is given twice daily at doses from 45 mg to 150 mg.

### 3.8. cAMeLot-1: Safety of Bleximenib in R/R Acute Leukemia with KMT2Ar and mNPM1

Bleximenib resulted in clinical DS in about 20% of patients, similar to rates reported with ziftomenib in the KOMET-001 trial (24%) and revumenib in phase II trials [29,31]. Grade 3 or higher DS occurred in 6% to 9% of patients, comparable to the 13% rate seen with revumenib in the mNPM1 AML cohort of the AUGMENT trial [29].

### 3.9. Enzomenib (DSP-5336)

Enzomenib is a new generation menin inhibitor with a unique chemical structure compared to other menin inhibitors, owing to its amide bond. The structure was chosen for further clinical development because of chemical properties such as low lipophilicity and basicity, which may lead to a low volume of distribution and low tissue accumulation. These properties were hypothesized to result in fewer extramedullary symptoms and potentially fewer DS, as observed with menin inhibitors [35,36,37].

### 3.10. Phase I/II Study of Enzomenib in R/R Acute Leukemia with KMT2Ar and mNPM1

In the first-in-human phase I trial, enzomenib showed no dose-limiting toxicity, including no QTc prolongation or dose-limiting DS. DS was reported in 10.7% of patients, about half the rate seen with other menin inhibitors (20–30%), and corticosteroid prophylaxis was not required. If confirmed, this may distinguish enzomenib from other agents. Early studies showed clinical response rates comparable to other menin inhibitors, with CCR rates of 30.4% in KMT2Ar and 47.1% in mNPM1 patients, and ORR of at least 50% in both groups. Enzomenib is also being studied in combination with venetoclax and azacitidine [35].

### 3.11. Phase I Enzomenib: Duration of Responses

The median time to objective response with enzomenib was 1.0 months in KMT2A-R patients and 1.9 months in mNPM1 patients, possibly due to its tissue distribution. With limited follow-up, the median DOR was not reached for KMT2Ar and was 7.0 months for mNPM1 [35].

Expansion cohorts are planned to further evaluate efficacy at the recommended phase 2 dose. Ongoing Studies combine enzomenib with azacitidine and venetoclax, and with gilteritinib for FLT3-mAML.

### 3.12. BN-104 and Additional Agents

Several additional menin inhibitors, including BN-104 [36], HMPL-506 [37], and icovamenib (BMF-219 [38], are in early clinical development. BN-104 has shown preliminary safety and efficacy in a phase 1/2 first-in-human study in Chinese patients with R/R AL [36]. The expanding number of menin inhibitors may enable sequencing strategies and selection based on resistance profiles, drug interactions, and combination compatibility [39].

## 4. Comparison with Standard Therapies

For newly diagnosed older adults with AML, ineligible for intensive chemotherapy, azacitidine plus venetoclax is the standard of care. The VIALE-A trial demonstrated higher CR rates (36.7% vs. 17.9%) and improved median OS (14.7 vs. 9.6 months) compared to azacitidine with placebo [40]. However, long-term outcomes remain limited, with 2-year OS below 40 percent [41]. Patients with NPM1 mutations or adverse-risk features continue to experience high relapse rates [42].

Patients with R/R KMT2A-rearranged AL have poor outcomes with standard salvage chemotherapy [43]. Median OS after the first relapse is 6 to 9 months and decreases with subsequent relapses. In heavily pretreated patients receiving third-line or later therapy, CR rates are typically 5 to 10 percent, and median survival is often limited to weeks or months. Even younger patients who tolerate intensive salvage regimens rarely achieve long-term survival without allo-SCT [44].

Venetoclax-based combinations are now commonly used as salvage therapy for NPM1-mutated R/R AML [45]. However, responses are often short-lived and variable. For patients with prior venetoclax exposure, further salvage options are limited [46].

Menin inhibitors have demonstrated significantly higher response rates than conventional salvage therapies in these molecularly defined populations [37]. Revumenib achieved a 23% CR/CRi rate in both KMT2A-rearranged and NPM1-mutated cohorts, including heavily pretreated patients, 75% of whom had prior venetoclax exposure [29]. High MRD negativity rates (61–75% among complete responders) and the ability to bridge about one-third of responders to transplantation show that menin inhibitors can improve long-term outcomes by providing deep and durable responses needed to improve. The median time to response of 1.9 to 2.8 months is clinically advantageous compared to longer response times with lower-intensity regimens. These data enable timely transplant planning for eligible patients.

## 5. Combination Strategy

Menin inhibitors have shown clinical activity as monotherapy in heavily pretreated populations, establishing proof of concept. However, optimal outcomes will likely require combination strategies, summarized in Table 1.

### 5.1. Incorporating Menin Inhibitors with Azacitidine and Venetoclax

Venetoclax resistance in AML is linked to upregulation of a KMT2A-like signature, marked by increased HOX and MEIS1 expression, which menin inhibition may counteract [47]. Menin inhibitors can repress HIF-1α-induced HDAC9, thereby synergizing with venetoclax in KMT2A-rearranged AML [48]. Several trials are evaluating menin inhibitors with venetoclax and azacitidine or decitabine/cedazuridine in newly diagnosed and R/R patients [49,50] (Table 2). In the BEAT AML trial, patients received a triplet regimen of azacitidine, venetoclax, and revumenib in the frontline setting [49]. This phase Ib/II study enrolled patients into two revumenib dose cohorts, combined with standard dosing for azacitidine and venetoclax. Patients who achieved remission continued triplet therapy until progression, transplant, or intolerance. Those who did not achieve a CR after 3 cycles were removed from the study due to futility. Among the 43 enrolled patients, the median age was 70 years, with 39.5% aged 75 or older. Co-occurring mutations included NRAS (21.4%), KRAS (11.9%), and FLT3-ITD (25.6%). One hematologic DLT was observed at dose level 1 (113 mg) during escalation. No other DLTs were reported, and no maximum tolerated dose was identified. The most common grade 1/2 treatment-emergent AEs were nausea, constipation, and QT prolongation. The 30- and 60-day mortality rates were both 7%. No grade 4 DS or QTc prolongation events were reported [15,16]. The CR rate was 67.4%, and the ORR was 88.4%. Patients with mNPM1 AML (*n* = 34) had an ORR of 85.3%, while those with KMT2Ar disease (*n* = 9) had an ORR of 100% [15,16].

The phase I/II SAVE trial at MD Anderson Cancer Center is evaluating an all-oral triplet of decitabine/cedazuridine, venetoclax, and revumenib in R/R AML. The reported ORR was 82%, with a combined CR and CRh of 48%. Sixty-five percent of patients were MRD-negative, and 39% proceeded to allo-SCT. The trial is now enrolling patients with newly diagnosed disease [50].

Bleximenib is being evaluated with venetoclax/azacitidine in newly diagnosed and R/R AML at a 100 mg twice-daily dose (NCT05453903) [51]. No dose-limiting toxicities (DLTs) were observed at this dose. Median platelet recovery ranged from 29 days in newly diagnosed patients to 38 days in R/R patients. The regimen was well tolerated, with no significant additional adverse events in the combination arm. The incidence of DS was lower with the venetoclax/azacitidine combination, with only 4% of patients experiencing grade 3 or higher DS. No significant QTc prolongation was reported; all events were grade 1 and did not require dose interruptions [51]. In a small cohort of newly diagnosed AML patients, early results of bleximenib plus chemotherapy have been promising. The combination of bleximenib with venetoclax and azacitidine yielded an ORR of 93.8% among 16 patients with mNPM1, with 75% achieving a CRc. In 4 patients with newly diagnosed AML and KMT2Ar, both the ORR and CRc were 75%. Among patients with R/R AML, 12 with mNPM1 achieved an ORR of 91.7% and a CRc of 66.7%, while 10 with KMT2Ar achieved an ORR of 70% and a CRc of 50% [51]. Patients on bleximenib/venetoclax/azacitidine received long-term treatment, with 65% of newly diagnosed patients still on therapy as of the data cutoff on 7 May 2025. Seven patients underwent subsequent allo-SCT: 2 from the newly diagnosed cohort and 5 from the R/R cohort.

KOMET-007 is an international, open-label, multi-part, multicohort phase I study evaluating ziftomenib with chemotherapy in patients with R/R or newly diagnosed AML characterized by KMT2A-R or mNPM1 [52,53]. Patients were enrolled in cohorts combining ziftomenib with either intensive (7 + 3) chemotherapy or less intensive chemotherapy (venetoclax plus azacitidine), based on overall fitness and performance status. The target ziftomenib dose from escalation was 600 mg, which was also the RP2D. The primary endpoints were DLT for phase Ia and CR and safety outcomes for phase Ib (NCT05735184). The average age of enrolled patients was 56 years. Patients with KMT2Ar AML had a median age of 43, while those with mNPM1 had a median age of 60. About 12% had therapy-related AML, which is more common in KMT2Ar patients. About one-third had mutations in FLT3 or IDH1/IDH2. Treatment with a venetoclax/azacitidine backbone alone would be expected to yield a CCR rate of about 20–30% [53]. The CR rate in this patient population was 23% with an ORR of 68%. Among patients with R/R mNPM1 AML who were not previously exposed to venetoclax (*n* = 8), the CCR rate was 75%, and the ORR was 100%. In comparison, among patients with R/R mNPM1 AML and prior venetoclax exposure (*n* = 14), the CCR rate was 36%, and the ORR was 50%. Among 11 patients (3 with mNPM1 and 8 with KMT2Ar) with prior menin-inhibitor experience, 2 with KMT2Ar disease responded to ziftomenib at 200 mg and 400 mg [53]. Six patients with R/R AML and mNPM1 who received ziftomenib plus venetoclax/azacitidine therapy received subsequent allo-SCT, with 1 patient on ziftomenib maintenance therapy. The 6-month OS was 77% for these patients. Among patients with KMT2Ar AML, 3 received subsequent allo-SCT, with 1 patient on ziftomenib maintenance therapy. The 6-month OS was 43% for these patients.

**Table 2:** Menin inhibitors trials with venetoclax and azacitidine or decitabine/cedazuridine.

Trial	Combination	Patient Population	Key Efficacy Outcomes	Notable Safety/ Tolerability	References
**BEAT AML Substudy**	Revumenib + Azacitidine + Venetoclax	Newly diagnosed older adults (age 60 or older) with NPM1-mutated or KMT2A-rearranged AML	CCR rate was 80 to 90%, with most achieving MRD negativity. Responses occurred rapidly, often within the first treatment cycle. At a median follow-up of 7 months, median OS was 15.5 months, and 1-year OS was 62.9 percent.	Acceptable tolerability profile	[49]
**SAVE Trial**	Revumenib + Decitabine/Cedazuridine + Venetoclax (all-oral triplet)	Relapsed/refractory AML	ORR: 82% CR/CRi: 48% MRD negativity: 88% (among CR/CRi responders) 6-month RFS: 59% 6-month OS: 74% Median DOR: Not yet reached	Well-tolerated all-oral regimen	[50]
**KOMET-007**	Ziftomenib + Venetoclax/Azacitidine	Relapsed/refractory with KMT2A-rearranged or NPM1-mutated AML	ORR: 50% CR/CRi: 25%	Differentiation syndrome resolved No ziftomenib-related QTc prolongation observed	[52,53]
**Bleximenib Phase 1b**	Bleximenib (100 mg BID) + Venetoclax + Azacitidine	Relapsed/refractory and newly diagnosed patients	Phase 1b dose established for phase 2 development	Acceptable safety profile at 100 mg BID dose	[51]

ORR: Overall Response Rate; CCR: Composite Complete Remission; CR: Complete Remission; CRi: Complete Remission with partial hematologic recovery; MRD: Minimal Residual Disease; RFS: Relapse-Free Survival; OS: Overall Survival; DOR: Duration of Response; BID: Twice daily.

### 5.2. Combination with Intensive Induction Chemotherapy

For patients eligible for intensive therapy, several studies are evaluating menin inhibitors with standard induction chemotherapy (7 + 3: cytarabine and daunorubicin) or FLAG-Ida (fludarabine, cytarabine, idarubicin) (Table 3).

In the KOMET-007 trial, early results indicate that ziftomenib did not significantly increase overall toxicity when added to the 7 + 3 regimen [52,53]. High rates of febrile neutropenia, nausea, vomiting, thrombocytopenia, and anemia were observed, as expected. Eighty-two patients developed DS, with only one grade 3 case, which was successfully managed. Two cases of QTc prolongation occurred in patients on other QTc-prolonging medications. Overall rates of sepsis and infection were low. The ziftomenib/7 + 3 combination showed promising clinical activity. Among 71 evaluable patients, 92% achieved a CCR, with a CR rate of 80% and an ORR of 94%. MRD negativity by central testing was achieved in 76% of evaluable patients, with a median time to MRD-negative CR of 4.5 weeks. Among patients with mNPM1 disease, 96% were still alive and on study after a median follow-up of 24.9 weeks. The median duration of CR and OS beyond 24 weeks was not reached. Two patients received a transplant, and three discontinued treatments due to relapse.

In the newly diagnosed KMT2Ar AML population treated with ziftomenib plus 7 + 3, with a median follow-up of 16 weeks, the median duration of CR was about 25 weeks, and the median OS has not been reached. Six patients underwent subsequent allo-SCT, with one receiving ziftomenib maintenance after transplant. Most patients with newly diagnosed KMT2Ar AML (88%) remain alive and on study. Although KMT2Ar leukemias exposed to prior therapy may be predisposed to cytopenias, there was no evidence of prolonged or delayed count recovery. Absolute neutrophil count and platelet recovery occurred within 29 to 32 days [52].

In the ALE1002 Phase 1b multicenter, dose-finding study (NCT05453903), patients received a standard 7 + 3 regimen with bleximenib. Among 24 patients, the ORR was 95.8%, CCR was 87.5%, and CR/CRh was 75%. The median duration of response was not reached [54].

### 5.3. FLT3 Inhibitor Combinations

About 30% of NPM1-mutated AML cases also have FLT3-ITD mutations [55]. Preclinical studies indicate potential synergy between menin and FLT3 inhibitors [56]. Clinical trials are evaluating combinations such as menin inhibitors with gilteritinib or quizartinib, and the enzomenib plus gilteritinib combination is being assessed.

### 5.4. Post-Transplant Maintenance

High relapse rates after allo-SCT in KMT2A-rearranged and high-risk NPM1-mutated AML make post-transplant maintenance with menin inhibitors a promising approach [57]. In the AUGMENT-101 trial, patients who achieved a CCR resumed revumenib after transplantation, and several restarted therapy. Further trials evaluating post-transplant maintenance are needed.

### 5.5. Emerging Combination Strategies: BCL-2 and CDK Inhibitors

Beyond currently evaluated combinations, preclinical evidence suggests potential synergy between menin inhibitors and other targeted therapies. BCL-2 inhibition with venetoclax has already demonstrated clinical efficacy in combination regimens (discussed in Section 5.1), but the mechanistic basis extends beyond simple additive effects. Menin inhibition downregulates MCL-1 and upregulates BAX, sensitizing cells to BCL-2 inhibition and overcoming venetoclax resistance mediated by HOX/MEIS1 upregulation [47,48].

CDK inhibitors represent another promising combination partner. CDK4/6 and CDK9 inhibitors have shown preclinical synergy with menin inhibitors in KMT2Ar models. Menin inhibition induces G1 cell cycle arrest and differentiation, which may be enhanced by CDK inhibition. CDK9 inhibitors, which suppress transcriptional elongation, may complement menin inhibitors’ effects on HOX gene transcription. Early-phase clinical trials combining menin inhibitors with selective CDK inhibitors are being planned.

Additional rational combinations under preclinical investigation include:

- DOT1L inhibitors: targeting a complementary epigenetic dependency in KMT2Ar leukemia
- BRD4 inhibitors: disrupting super-enhancer-driven gene expression.
- HDAC inhibitors: enhancing chromatin accessibility and differentiation.

The expanding portfolio of menin inhibitors with distinct safety profiles (Table 4) may enable tailored combination strategies based on overlapping toxicities and mechanistic synergies.

## 6. Challenges in Patient Selection and Resistance Mechanisms

Effective use of menin inhibitors depends on accurately identifying patients with KMT2A rearrangements or NPM1 mutations. KMT2A rearrangements can be detected by several methods, including fluorescence in situ hybridization (FISH) targeting the KMT2A locus, reverse transcriptase polymerase chain reaction (RT-PCR) to detect specific fusion transcripts, and next-generation sequencing (NGS) panels that detect both rearrangements and point mutations [58,59,60]. Long-read NGS technologies are increasingly used to identify occult genomic rearrangements and complex RNA variants that may be missed by conventional methods [60] (UPDATED: Winters et al. 2024, PMID: 39823827).

For revumenib, FDA approval requires confirmation of KMT2A translocations using an FDA-authorized test. NPM1 mutations are typically detected by NGS or PCR of the terminal exon, with “susceptible mutations” defined in the labeling.

Most institutional molecular diagnostic platforms can detect NPM1 mutations, but fully characterizing KMT2A rearrangements is more challenging. With over 120 potential fusion partners, targeted PCR may miss rare rearrangements [61]. FISH is reliable for detection but does not identify the fusion partner. RNA-based NGS offers the most comprehensive characterization, though it may not be widely available. Partial tandem duplications of KMT2A (KMT2A-PTD) are excluded from the revumenib indication because they lack the menin-binding domain required for drug activity [18]. This highlights the need for accurate molecular characterization.

## 7. Mechanisms of Acquired Resistance

The primary resistance mechanism is the development of somatic MEN1 mutations at sites critical for menin inhibitor binding. Perner et al. found that patients treated with revumenib acquired MEN1 mutations, such as M327I, M327V, G331R, G331D, and T349M, during disease progression [62]. Crystallographic and computational studies showed that these mutations alter amino acid residues in the drug-binding pocket of menin. The resulting steric clashes block inhibitor binding but do not disrupt the menin-KMT2A interaction. This allows the KMT2A-menin complex to maintain oncogenic activity on chromatin despite treatment.

MEN1 mutations may develop rapidly, sometimes within two therapy cycles (about 8 weeks). In the AUGMENT-101 trial, about 40% of patients with adequate DNA samples and treatment exposure developed detectable MEN1 mutations before or during progression [63]. Droplet digital PCR can detect low-level MEN1 mutant alleles before morphologic relapse, suggesting that serial monitoring may allow early identification of resistance. Base editor screening studies show that MEN1 mutations confer varying resistance to menin inhibitors. M327 alterations confer resistance to all current inhibitors, whereas other mutations exhibit selective resistance. For example, some mutations confer high resistance to enzomenib but minimal resistance to bleximenib. These results suggest that sequential use of different menin inhibitors may be feasible based on resistance profiles. Not all acquired resistance cases involve MEN1 mutations. In approximately 60% of patients with resistance in clinical trials, no MEN1 mutations were detected [62]. RNA sequencing of these MEN1 wildtype-resistant cases showed distinct transcriptional reprogramming, including reduced expression of menin-KMT2A target genes such as MEIS1 and HOX, and increased expression of myeloid differentiation genes [64,65].

**Figure 3:**
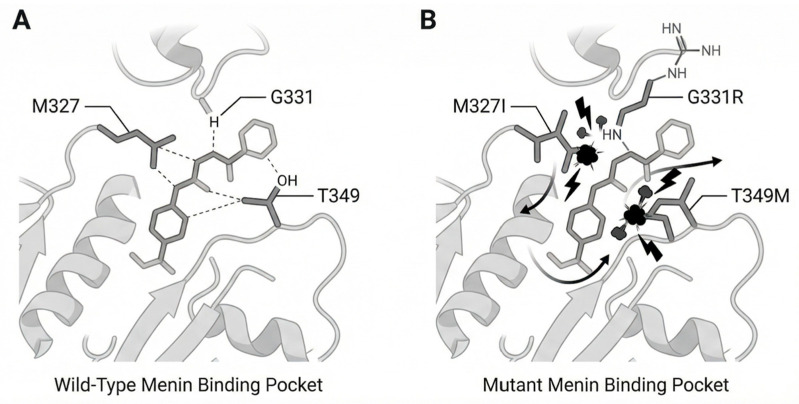
Wild-Type vs. Mutant Menin-Inhibitor Interaction. Panel (**A**) Wild type menin binding pocket (M327, G331, T349) with inhibitor. Panel (**B**) Mutant menin (M327I, G331R, T349M) showing steric clash preventing inhibitor binding.

The molecular mechanisms underlying this non-genetic resistance are still being studied. Potential factors include epigenetic reprogramming, activation of HOXA/MEIS1-independent survival pathways, clonal evolution with reduced dependency on menin-KMT2A signaling, and microenvironmental influences that support drug-tolerant persistence [47,66] (Figure 3).

## 8. Strategies to Overcome Resistance

### 8.1. Next-Generation Menin Inhibitors

Structure-guided drug design has produced second-generation menin inhibitors effective against common MEN1 resistance mutations [16]. Blenreximab is active against specific MEN1 mutations (M327I, T349M) that confer resistance to vemurafenib, showing that alternative drug binding can overcome these mutations [25]. Not all resistance mutations are addressed.

### 8.2. Combination Approaches to Prevent Resistance

Early and sustained combination therapy may delay or prevent resistance by achieving deeper remissions with higher rates of MRD negativity; reducing the tumor burden from which resistant clones can emerge; providing complementary mechanisms of action that prevent the outgrowth of resistant populations; and high complete remission rates and MRD negativity with triplet combinations of menin inhibitor, azacitidine, and venetoclax support this strategy [67].

### 8.3. Sequential Therapy Based on Resistance Profiling

As resistance profiles for MEN1 inhibitors become clearer, molecular monitoring to identify specific MEN1 mutations at progression may guide the selection of alternative inhibitors [68]. This sequential approach needs prospective validation but may extend patient benefit.

### 8.4. Bridging to Allo-SCT

For eligible patients, achieving remission with menin inhibitors to enable allo-SCT remains the most definitive strategy to overcome resistance. In clinical trials, 30 to 40 percent of responders who proceeded to transplant were potentially cured, even after relapse or prior treatment failure [18,29,69].

### 8.5. Monitoring and Surveillance

Prospective monitoring of MEN1 mutation status is recommended for patients on menin inhibitors, especially those with delayed, incomplete, or declining responses. Serial monitoring with sensitive methods such as droplet digital PCR can detect emerging resistance mutations early and allow timely intervention [63,68].

## 9. Safety Considerations

Myelosuppression is expected due to the induction of leukemic blast differentiation [5,29,53,69]. Common cytopenias include thrombocytopenia, neutropenia, and anemia, typically managed with supportive care like transfusions and growth factors. The duration of cytopenias depends on disease burden, prior therapies, and remission status.

Nausea, vomiting, diarrhea, and decreased appetite are common, affecting 20–40% of patients [5,29,53,69]. These symptoms are usually mild to moderate and managed with supportive medications.

DS is a significant adverse event with menin inhibitors, occurring in about 10–15% of patients [39]. DS likely results from rapid differentiation and maturation of leukemic blasts, like the syndrome seen with all-trans retinoic acid in acute promyelocytic leukemia or with IDH inhibitors. Symptoms include unexplained fever, weight gain, peripheral edema, shortness of breath, pleural or pericardial effusions, pulmonary infiltrates on imaging, hypotension, renal dysfunction, and a rapidly increasing white blood cell count. Early recognition and prompt initiation of corticosteroids, such as dexamethasone 10 mg twice daily or equivalent, are critical. Most cases respond well and do not require stopping treatment. In the AUGMENT-101 trial, differentiation syndrome was manageable in most patients [29]. Patients who experienced DS had higher ORRs (75% vs. 42%), suggesting a correlation with treatment effect [16]. Prophylactic corticosteroids are not routinely used but may be considered for high-risk patients. Careful monitoring during the first 2–4 weeks of therapy, when DS typically occurs, allows early intervention.

QTc interval prolongation has been observed with some menin inhibitors, especially revumenib, which requires ECG monitoring [16]. Revumenib prescribing information recommends baseline ECG and electrolyte assessment, with periodic monitoring during treatment. Patients with a baseline QTc greater than 470 msec or other risk factors for QT prolongation need more intensive monitoring and dose adjustments as needed.

Not all menin inhibitors have the same effect on QTc. Ziftomenib and bleximenib have shown minimal or no QTc prolongation in trials [16]. This may influence drug selection, particularly for combination strategies or for patients with cardiac risk factors.

## 10. Emerging Opportunities

Promising research directions include several areas. KMT2A-rearranged infant leukemia remains a challenging population with poor outcomes and limited treatment options [26]. Expanding menin inhibitor development to younger pediatric patients, with age-adjusted dosing and safety monitoring, could significantly improve outcomes in this group.

While most development has focused on acute myeloid leukemia, KMT2A rearrangements also occur in ALL, particularly in infants and young children [70]. Preclinical data support the activity of menin inhibitors in KMT2A-rearranged ALL, and clinical trials are underway.

NPM1 mutations and, at times, KMT2A alterations are present in myelodysplastic syndromes and related disorders [71]. Investigating menin inhibitors in these settings may broaden their application.

Beyond current combinations, pairing menin inhibitors with emerging therapies such as immune checkpoint inhibitors, CD47 inhibitors, CAR T-cell therapy, and new targeted agents should be explored [72]. Downregulation of HOXA9 and other stemness factors by menin inhibitors may enhance tumor sensitivity to immunotherapies.

Integrating comprehensive genomic, transcriptomic, and proteomic profiling at baseline and during therapy may enable precision medicine, optimize patient selection, predict resistance, and guide treatment sequencing [73].

## 11. Expert Opinion

Revumenib’s FDA approvals (November 2024 for KMT2A-rearranged, October 2025 for NPM1 mutated R/R acute leukemia) validate menin inhibition as the first molecularly targeted therapy for these historically difficult-to-treat populations. In September 2025, revumenib was added to the NCCN Clinical Practice Guidelines in Oncology for AML as a Category 2A recommended treatment option for R/R NPM1-mutated AML, providing guidance to community practitioners [74]. After two decades of research, we finally have a precision medicine approach that addresses the fundamental biology driving these leukemias.

Despite these advances, several important questions remain (Table 5 and Table 6). First of all, the question is the optimal positioning in treatment sequence: should menin inhibitors be reserved for R/R disease, or included in frontline therapy for patients with targetable alterations? High response rates with combinations such as intensive chemotherapy or azacitidine-venetoclax suggest potential frontline use, but randomized trials are needed to confirm benefit and guide practice.

Second question: who benefits most? Our priority populations are: R/R KMT2A-rearranged patients (especially young adults/children), 53% of ORR, with 26% achieving transplant-eligible MRD-negative remissions; R/R NPM1-mutated patients who have exhausted standard options (47% ORR, enabling 17% to proceed to potentially curative transplant); newly diagnosed unfit patients in clinical trials (triplet regimens—menin inhibitor + azacytidine/venetoclax—achieving 80–90% MRD negativity-transformative for elderly patients who traditionally do poorly). Unlike conventional chemotherapy, menin inhibitors induce differentiation rather than cytotoxic kill. This mechanism explains high rates of MRD negativity (70–90% in triplet regimens), durable responses enabling successful transplant bridge (75% post-allo-SCT remission maintenance), unique toxicity profile (DS, manageable gastrointestinal side effects). The depth of molecular response matters. The transplant colleagues consistently report better outcomes when patients achieve MRD-negative status pre-allo-SCT, and menin inhibitors deliver this in populations where it was previously rare. 30–40% of responders who reach transplant achieve potential cure. Every responder deserves prompt transplant evaluation, because menin inhibitors buy time but transplant offers cure.

Moreover, is upfront combination therapy better than using menin inhibitors sequentially after standard therapy? While combinations may prevent resistance and achieve deeper remissions, they also increase toxicity and cost. Combining menin inhibitors with other targeted therapies is something that is being investigated now, but it is something with which we need more experience. Are we going to have specific algorithms for patients with co-occurring FLT3, IDH, and NPM1 mutations? Are we going to be administering complicated regimens like induction chemotherapy with 7 + 3, and a FLT3 inhibitor, an IDH inhibitor, and a menin inhibitor in attempts to induce patients into the deepest remission possible? Perhaps then removing some of those drugs to transition to maintenance therapy?

Another question is the duration of therapy. The optimal duration of therapy for patients in CR is unclear. Should treatment continue until progression, for a set period, or as maintenance after consolidation or transplantation? Prospective studies are needed to balance relapse prevention, toxicity, and quality of life.

Furthermore, post-transplant maintenance. Due to high relapse rates after allo-SCT in these high-risk subtypes, post-transplant maintenance is a promising strategy. Data from dedicated trials are needed to guide duration and management, including graft-versus-host disease. We assume that maintenance therapy after allo-SCT is beneficial, but we think that needs to be proven in a randomized study.

Biomarkers: can we identify patients most likely to respond to or develop resistance to menin inhibitors? Baseline gene expression, co-mutations, and early MRD dynamics may help stratify patients. Comprehensive molecular profiling at baseline and during treatment will be essential in future trials.

Another dark side is the resistance prevention and management. Identifying MEN1 mutations as a primary resistance mechanism has enabled the design of new drugs and the development of sequential therapy strategies. Can next generation menin inhibitors active against resistant mutations prevent or delay resistance? Can combination approaches eliminate resistant clones before they emerge? Serial molecular monitoring and early intervention may be effective. The esistance challenge MEN1 mutations emerge in ~40% of patients, typically by cycle 2–4 (8–12 weeks). Clonal heterogeneity represents a fundamental challenge in the treatment of MLL1r and NPM1-mutated AML, with significant implications for therapeutic resistance both at diagnosis and following targeted intervention. Two principal mechanisms underlie treatment failure in these molecularly defined subsets: chromosomal instability-driven clonal evolution and the activation of bypass pathways that operate independently of the canonical Menin–MLL1/HOX–MEIS axis. Chromosomal instability is an inherent feature of MLL1r AML, arising from the disruption of normal chromatin regulation and genomic maintenance by MLL1 fusion proteins. This genomic instability generates a diverse clonal architecture characterized by chromosomal breaks, aneuploidy, and structural variations that provide a substrate for adaptive resistance. The polyclonal nature of MLL1r leukemia enables the rapid selection of pre-existing resistant subclones harboring secondary mutations in genes such as FLT3, RAS pathway components, and TP53. Additionally, acquired chromosomal alterations, including trisomy 8 and monosomy 7, have been documented during menin inhibitor therapy, further compounding resistance. This dynamic clonal evolution necessitates rational combination strategies, such as concurrent menin and FLT3 inhibition in patients with FLT3-ITD co-mutations, as well as the potential exploitation of replication stress through DNA damage response inhibitors targeting PARP or ATR pathways. In NPM1-mutated AML, resistance to menin inhibition extends beyond perturbation of the HOX–MEIS axis, reflecting the pleiotropic effects of mutant NPM1 on cellular homeostasis. While initial leukemic dependency on HOX/MEIS-mediated transcriptional programs renders these cells sensitive to menin inhibitors, alternative survival pathways rapidly emerge. Cytoplasmic mislocalization of mutant NPM1 results in constitutive NF-κB activation, providing survival signals independent of HOX gene expression. Furthermore, NPM1 mutations enhance ribosomal biogenesis and dysregulate nucleocytoplasmic transport, creating dependencies that bypass menin-mediated chromatin regulation. The co-occurrence of mutations in epigenetic modifiers (DNMT3A, IDH1/2, TET2) and FLT3-ITD establishes parallel proliferative and epigenetic programs that sustain leukemic growth despite menin inhibition. Consequently, effective therapeutic strategies must employ upfront combination approaches, including menin inhibitors with BCL-2 inhibitors such as venetoclax, FLT3 inhibitors, or XPO1 inhibitors to restore nuclear NPM1 localization and tumor suppressor function. These observations underscore the necessity of anticipating resistance mechanisms through rational polytherapy rather than sequential monotherapy, given the remarkable adaptability conferred by clonal diversity in both MLL1r and NPM1-mutated AML.

Finally, menin inhibitors are associated with DS, which can have serious consequences for our patients. DS usually occurs in the first cycle with the initiation of menin inhibitor therapy. The median time to presentation of DS can be as short as 3–5 days and up to 8–10 days after initiation of therapy. Treatment and prophylaxis of DS can be important. Across the menin inhibitor trials, we can see DS rates of 10.7% to 28%. Grade 3 DS with mitigation strategies has been reduced to approximately 12% to 16%. However, management really involves early recognition of patients at high risk of disease. WBC counts must be brought under 20,000 at the time of menin inhibitor initiation. Cytoreduction with hydroxyurea or cytarabine can achieve lower WBC counts. Patients who need cytoreduction prior to therapy should be put on prophylactic steroids with the initiation of the menin inhibitor. Also, if mitigation and supportive care strategies, including management of tumor lysis and DIC, do not quickly resolve in DS, then the menin inhibitor should be held until better clinical control is achieved. With appropriate mitigation strategies, DS is a manageable side effect. Although DS can be fatal in patients with AML, we do not withhold treatment of this highly curable cancer as a result.

**Figure 4:**
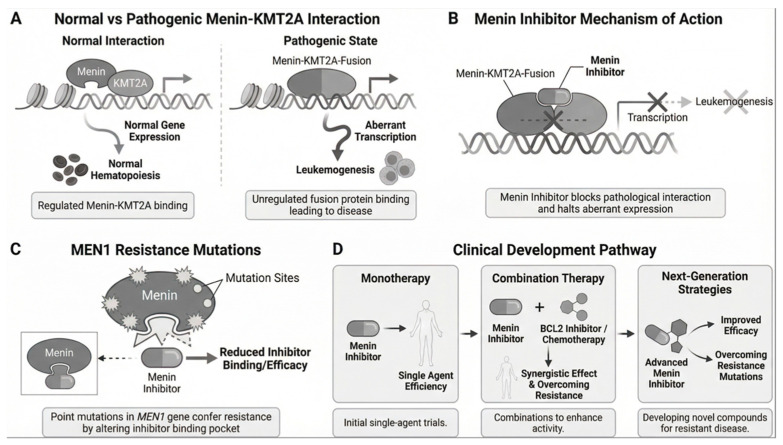
Integrated Mechanistic and Clinical Development Diagram. Panel (**A**) Normal vs. Pathogenic Menin-KMT2A Interaction. Panel (**B**) Menin Inhibitor Mechanism of Action. Panel (**C**) MEN1 Resistance Mutations. Panel (**D**) Clinical Development Pathway (Monotherapy → Combinations → Next-Generation Strategies).

Figure 4 provides an integrated overview of menin inhibitor development and resistance mechanisms. Panel A illustrates the normal menin-KMT2A interaction in wild-type hematopoiesis and the pathogenic menin-KMT2A fusion protein interaction in KMT2Ar leukemia, both of which depend on the menin-binding domain. Panel B demonstrates how menin inhibitors competitively disrupt this interaction, leading to HOX gene downregulation and myeloid differentiation. Panel C shows the emergence of MEN1 resistance mutations (M327I/V, G331R/D, T349M) that prevent inhibitor binding while maintaining menin-KMT2A interaction. Panel D maps the clinical development pathway from monotherapy trials (AUGMENT-101, KOMET-001) through combination strategies (with venetoclax/azacitidine, intensive chemotherapy) to next-generation approaches addressing resistance.

## 12. Conclusions

Menin inhibitors represent a significant advancement in the treatment of KMT2A-rearranged and NPM1-mutated ALs by targeting the core molecular drivers of these diseases. Key achievements include durable CRs with high rates of MRD negativity in patients who have no alternative standard treatments, establishing menin inhibitors as a valuable option. Disease control that enables allo-SCT in about one-third of responders offers a potential cure. Extending clinical benefit to NPM1-mutated AML broadens the impact, potentially benefiting up to 40% of AML patients when both subtypes are considered. While on-target effects such as myelosuppression and differentiation syndrome can occur, the toxicity profile is manageable with appropriate monitoring and supportive care, allowing for extended therapy.

The development of menin inhibitors demonstrates how molecular insights can be effectively translated into clinical therapies. The progression from discovering KMT2A rearrangements and studying menin binding to structure-guided drug design and clinical validation exemplifies precision oncology. Rapid adoption into treatment guidelines and clinical practice after regulatory approval highlights the field readiness to implement targeted therapies with proven benefits.

Menin inhibitors have the potential to transform treatment for patients with KMT2A-rearranged and NPM1-mutated ALs. Although challenges remain such as resistance and determining optimal use, there is a strong foundation for further refinement and expansion. As clinical experience grows, long-term data are collected, and strategies to address resistance are explored, menin inhibitors are likely to become a cornerstone of precision therapy for these subtypes. Their success provides a roadmap for targeting other protein–protein interactions once considered “undruggable.” For patients with KMT2A-rearranged or NPM1-mutated leukemia, menin inhibitors offer renewed hope, turning once-fatal diagnoses into treatable conditions with real prospects for long-term survival.

## Figures and Tables

**Table 1:** Menin inhibitors: rationale for a combination strategy.

Rationale	Mechanism/Benefit
Mechanistic synergy	Menin inhibition downregulates HOX genes and MEIS1, which are linked to resistance to venetoclax and other targeted therapies. This epigenetic regulation supports combination approaches.
Non-overlapping toxicities	Menin inhibitors have distinct mechanisms and adverse event profiles, allowing combination with chemotherapy, hypomethylating agents, and other targeted therapies without significant additive toxicities.
Prevention of resistance	Combination approaches may delay or prevent the emergence of resistance more effectively than sequential monotherapy.

**Table 3:** Menin inhibitors in combination with standard induction chemotherapy.

Trial	Combination	Patient Population	Key Efficacy Outcomes	Notable Safety/Tolerability	Reference
**KOMET-007**	Ziftomenib + 7 + 3	Newly diagnosed high-risk with KMT2A-rearranged or NPM1-mutated AML	Composite CR rates: 83% to 100% across dose levels	Data not yet reported	[52]
**ALE1002**	Bleximenib + 7 + 3	Newly diagnosed patients eligible for intensive chemotherapy (*n* = 28)	ORR: 95% Composite CR rate: 86%	No cases of differentiation syndrome No QTc prolongation reported	[54]

7 + 3: Standard induction chemotherapy (7 days cytarabine + 3 days anthracycline); ORR: Overall Response Rate; CR: Complete Remission; Composite CR: Includes CR, CRi (CR with incomplete count recovery), and/or CRh (CR with partial hematologic recovery).

**Table 4:** Comparison of Menin Inhibitor Monotherapy Trials.

Agent	Trial	N	ORR (%)	CCR (%)	QTc Prolongation	DS (Grade ≥ 3)
Revumenib	AUGMENT-101 (KMT2Ar)	104	64	23	Grade 3: 19% Grade 4: 2%	13%
Revumenib	AUGMENT-101 (NPM1m)	84	46.9	23.4	Grade 3: 19% Grade 4: 2%	13%
Ziftomenib	KOMET-001	112	NR	25	No significant	13%
Bleximenib	cAMeLot-1	NR	NR	NR	Minimal	6–9%
Enzomenib	Phase I/II	56	≥50	KMT2Ar: 30.4 NPM1m: 47.1	None	<5%

Key safety distinctions: QTc Prolongation—Revumenib requires intensive cardiac monitoring; ziftomenib, bleximenib, and enzomenib show minimal QTc effects. DS—Enzomenib shows the lowest rate (10.7%). Tolerability—Ziftomenib had only 3% discontinuation due to treatment-related AEs.

**Table 5:** Menin inhibitors: integration into clinical practice.

**Treatment Algorithm for KMT2A-Rearranged AML**				
**Clinical Setting**	**Potential Scenario**		**Key Considerations**	
**Newly Diagnosed, Fit for Intensive Therapy**	Enroll in clinical trials combining menin inhibitor with intensive chemotherapy (7 + 3) Administer standard intensive induction Consolidation with allo-SCT when feasible		Bleximenib + 7 + 3 trial (ALE1002 Arm C) Ziftomenib + 7 + 3 trial Bridge to transplant in first remission	
**Newly Diagnosed, Unfit for Intensive Therapy**	**Preferred**: Clinical trial with menin inhibitor + azacitidine + venetoclax **Alternative**: Standard azacitidine + venetoclax, add menin inhibitor at progression		ALE1002 trial (bleximenib + azacitidine + venetoclax) BEAT AML trial (revumenib + azacitidine + venetoclax) Consider reduced-intensity conditioning allo-SCT for select patients High response rates (≥80%) reported in de novo triplet trials	
**Relapsed/Refractory**	**Standard**: Revumenib monotherapy (FDA-approved) **Alternative**: Clinical trials with combination regimens		FDA-approved for R/R KMT2Ar acute leukemia (Nov 2024) NCCN Category 2A recommendation SAVE trial (revumenib + decitabine/cedazuridine. + venetoclax): 82% ORR Bridge to allo-SCT in responders (75% maintain remission post-transplant)	
**Treatment Algorithm for NPM1-Mutated AML**				
**Clinical Setting**	**Potential Scenario**		**Key Considerations**	
**Newly Diagnosed, Fit for Intensive Therapy**	Standard intensive chemotherapy ± FLT3 inhibitor (if FLT3-mutated) Enroll in clinical trials investigating menin inhibitor combinations		Frontline menin inhibitor trials enrolling Role in consolidation/maintenance under investigation BEAT AML consortium studying revumenib + azacitidine + venetoclax in older adults	
**Newly Diagnosed, Unfit for Intensive Therapy**	Standard azacitidine + venetoclax Consider clinical trial with menin inhibitor triplet combination		Multiple trials investigating menin inhibitor + hypomethylating agent + venetoclax High MRD-negative rates (70–90%) in trials Consider as bridge to reduced-intensity allo-SCT	
**Relapsed/Refractory**	**Standard:** Revumenib monotherapy (FDA-approved Oct 2025) **Alternatives:** Clinical trials with combination regimens Venetoclax-based regimens (if not previously received)		FDA-approved for R/R NPM1m AML (24 October 2025) NCCN Category 2A (added Sept 18, 2025) CCR rate: 23.4%, ORR: 46.9% Median DOR: 4.5 months Bridge to allo-SCT in responders Ziftomenib also NCCN Category 2A	
**Post-Transplant Strategies: potential scenario**				
**Strategy**	**Patient Selection**	**Evidence**		**Outcomes**
**Post-Transplant Maintenance**	Patients achieving remission with menin inhibitor Underwent allo-SCT Good post-transplant performance status	9 patients from AUGMENT-101 resumed revumenib (8 post-allo-SCT, 1 post-stem cell boost) Duration: 23–588 days 5 patients ongoing at data cutoff		6/9 maintained CR 5 achieved/sustained MRD negativity Long-term responses (>1 year) in 3 patients Conversion to MRD-negative status observed
**Transplant as Consolidation**	Responders to menin inhibitor therapy Medically eligible for allo-SCT	Phase 1: 12 transplanted, 9 in remission at cutoff (75%) Phase 2 KMT2Ar: Multiple patients proceeded to allo-SCT Phase 2 NPM1m: 5/30 responders (16.7%) proceeded to allo-SCT		30–40% of all responders achieve potentially curative outcomes via transplant Durable remissions (>6 months) in majority of transplanted patients

**Table 6:** Monitoring Recommendations During Menin Inhibitor Therapy.

Parameter	Method	Frequency	Rationale
**MEN1 Mutation Status**	Droplet digital PCR (ddPCR) Cell-free DNA NGS (e.g., MSK-ACCESS-MEN1)	Baseline After 2 cycles (~8 weeks) At response decline At progression	Detect resistance mutations at <1% VAF Mutations emerge ~2 cycles (8 weeks) Enable early intervention before morphologic relapse Guide sequential menin inhibitor selection
**MRD Assessment**	Flow cytometry Molecular methods (NPM1 qPCR)	After cycle 1 At response assessment Every 2–3 cycles	MRD negativity achieved in 70–90% of responders Prognostic for duration of response Guide consolidation decisions
**Differentiation Syndrome**	Clinical assessment CBC with differential Inflammatory markers	Weekly during first 2 cycles At each visit thereafter	Class effect of menin inhibitors Can be life-threatening if unrecognized Early steroid intervention critical

## Data Availability

Not applicable.

## References

[1] Han X., Yun Z., Liu Z., Si Y., Tian S., Zhang Y., Qi Y., Xue C., Cui M., Wen X. (2025). Global, regional, and national burden of acute leukemia and its risk factors from 1990 to 2021 and predictions to 2040: Findings from the global burden of disease study 2021. Biomed. Eng. Online.

[2] Guarnera L., D’addona M., Bravo-Perez C., Visconte V. (2024). *KMT2A* Rearrangements in Leukemias: Molecular Aspects and Therapeutic Perspectives. Int. J. Mol. Sci..

[3] Falini B., Martelli M.P., Bolli N., Sportoletti P., Liso A., Tiacci E., Haferlach T. (2011). Acute myeloid leukemia with mutated nucleophosmin (*NPM1*): Is it a distinct entity?. Blood.

[4] Döhner H., Wei A.H., Appelbaum F.R., Craddock C., DiNardo C.D., Dombret H., Ebert B.L., Fenaux P., Godley L.A., Hasserjian R.P. (2022). Diagnosis and management of AML in adults: 2022 recommendations from an international expert panel on behalf of the ELN. Blood.

[5] Issa G.C., Bidikian A., Venugopal S., Konopleva M., DiNardo C.D., Kadia T.M., Borthakur G., Jabbour E., Pemmaraju N., Yilmaz M. (2023). Clinical outcomes associated with *NPM1* mutations in patients with relapsed or refractory AML. Blood Adv..

[6] Krivtsov A.V., Armstrong S.A. (2007). *MLL* translocations, histone modifications and leukaemia stem-cell development. Nat. Rev. Cancer.

[7] Issa G.C., Ravandi F., DiNardo C.D., Jabbour E., Kantarjian H.M., Andreeff M. (2021). Therapeutic implications of menin inhibition in acute leukemias. Leukemia.

[8] Zhang R., Huang H., Zhang Y., Xia Y., Huang J., Jiang C., Wang L., Lu H., Pan Z., Wang G. (2025). Outcomes of acute myeloid leukemia with *KMT2A* (*MLL*) rearrangement: A multicenter study of TROPHY group. Blood Cancer J..

[9] Richard-Carpentier G., Kantarjian H.M., Tang G., Yin C.C., Khoury J.D., Issa G.C., Haddad F., Jain N., Ravandi F., Short N.J. (2021). Outcomes of acute lymphoblastic leukemia with *KMT2A* (*MLL*) rearrangement: The MD Anderson experience. Blood Adv..

[10] Thorsteinsdottir U., Kroon E., Jerome L., Blasi F., Sauvageau G. (2001). Defining roles for HOX and MEIS1 genes in induction of acute myeloid leukemia. Mol. Cell. Biol..

[11] Grembecka J., He S., Shi A., Purohit T., Muntean A.G., Sorenson R.J., Showalter H.D., Murai M.J., Belcher A.M., Hartley T. (2012). Menin-MLL inhibitors reverse oncogenic activity of *MLL* fusion proteins in leukemia. Nat. Chem. Biol..

[12] Krivtsov A.V., Evans K., Gadrey J.Y., Eschle B.K., Hatton C., Uckelmann H.J., Ross K.N., Perner F., Olsen S.N., Pritchard T. (2019). A Menin-MLL Inhibitor Induces Specific Chromatin Changes and Eradicates Disease in Models of *MLL*-Rearranged Leukemia. Cancer Cell.

[13] Uckelmann H.J., Kim S.M., Wong E.M., Hatton C., Giovinazzo H., Gadrey J.Y., Krivtsov A.V., Rücker F.G., Döhner K., McGeehan G.M. (2020). Therapeutic targeting of preleukemia cells in a mouse model of *NPM1* mutant acute myeloid leukemia. Science.

[14] Thomas X. (2024). Small Molecule Menin Inhibitors: Novel Therapeutic Agents Targeting Acute Myeloid Leukemia with *KMT2A* Rearrangement or *NPM1* Mutation. Oncol. Ther..

[15] Cantilena S., AlAmeri M., Che N., Williams O., de Boer J. (2024). Synergistic Strategies for *KMT2A*-Rearranged Leukemias: Beyond Menin Inhibitor. Cancers.

[16] Dali S.A., Al-Mashdali A.F., Kalfah A., Mohamed S.F. (2025). Menin inhibitors in *KMT2A*-rearranged and *NPM1*-mutated acute leukemia: A scoping review of safety and efficacy. Crit. Rev. Oncol. Hematol..

[17] Yang X., Huang R. (2025). Menin inhibitors in *KMT2A*-rearranged leukemia: Mechanistic insights, clinical trial progress, and potential of combination therapies. Leuk. Res..

[18] Issa G.C., Aldoss I., DiPersio J., Cuglievan B., Stone R., Arellano M., Thirman M.J., Patel M.R., Dickens D.S., Shenoy S. (2023). The menin inhibitor revumenib in *KMT2A*-rearranged or *NPM1*-mutant leukaemia. Nature.

[19] FDA Approves Revumenib for Relapsed or Refractory Acute Leukemia with a KMT2A Translocation. https://www.fda.gov/drugs/resources-information-approved-drugs/fda-approves-revumenib-relapsed-or-refractory-acute-leukemia-kmt2a-translocation.

[20] GlobeNewswire Syndax Announces FDA Approval of Revuforj^®^ (Revumenib) in Adult and Pediatric Patients with Relapsed or Refractory *NPM1* Mutated Acute Myeloid Leukemia. https://ir.syndax.com/news-releases/news-release-details/syndax-announces-fda-approval-revuforjr-revumenib-adult-and.

[21] Zhang Y., Mittal A., Reid J., Reich S., Gamblin S.J., Wilson J.R. (2015). Evolving Catalytic Properties of the *MLL* Family SET Domain. Structure.

[22] Schurer A., Glushakow-Smith S.G., Gritsman K. (2025). Targeting chromatin modifying complexes in acute myeloid leukemia. Stem Cells Transl. Med..

[23] Zehtabcheh S., Soleimani Samarkhazan H., Asadi M., Zabihi M., Parkhideh S., Mohammadi M.H. (2025). Insights into *KMT2A* rearrangements in acute myeloid leukemia: From molecular characteristics to targeted therapies. Biomark. Res..

[24] Chapsal B.D., Kimbrough J.R., Bester S.M., Bergstrom A., Backos D.S., Campos B., McDonald M.G., Abrahamsen R., Allen A.C., Barbour P.M.D. (2024). Design of Potent Menin-*KMT2A* Interaction Inhibitors with Improved In Vitro ADME Properties and Reduced hERG Affinity. ACS Med. Chem. Lett..

[25] Ray J., Clegg B., Grembecka J., Cierpicki T. (2024). Drug-resistant menin variants retain high binding affinity and interactions with *MLL1*. J. Biol. Chem..

[26] Cuglievan B., Kantarjian H., Rubnitz J.E., Cooper T.M., Zwaan C.M., Pollard J.A., DiNardo C.D., Kadia T.M., Guest E., Short N.J. (2024). Menin inhibitors in pediatric acute leukemia: A comprehensive review and recommendations to accelerate progress in collaboration with adult leukemia and the international community. Leukemia.

[27] Uckelmann H.J., Haarer E.L., Takeda R., Wong E.M., Hatton C., Marinaccio C., Perner F., Rajput M., Antonissen N.J.C., Wen Y. (2023). Mutant *NPM1* Directly Regulates Oncogenic Transcription in Acute Myeloid Leukemia. Cancer Discov..

[28] Dhiman S., Dhillon V., Balasubramanian S.K. (2024). Targeting Menin in Acute Myeloid Leukemia: Therapeutic Advances and Future Directions. Cancers.

[29] Arellano M.L., Thirman M.J., DiPersio J.F., Heiblig M., Stein E.M., Schuh A.C., Žučenka A., de Botton S., Grove C.S., Mannis G.N. (2025). Menin inhibition with revumenib for *NPM1*-mutated relapsed or refractory acute myeloid leukemia: The AUGMENT-101 study. Blood.

[30] Shimony S., Stahl M., Stone R.M. (2025). Acute Myeloid Leukemia: 2025 Update on Diagnosis, Risk-Stratification, and Management. Am. J. Hematol..

[31] Wang E.S., Montesinos P., Foran J., Erba H., Rodríguez-Arbolí E., Fedorov K., Heiblig M., Heidel F.H., Altman J.K., Baer M.R. (2025). Ziftomenib in Relapsed or Refractory *NPM1*-Mutated, A.M.L. J. Clin. Oncol..

[32] Smith C.C., Levis M.J., Perl A.E., Hill J.E., Rosales M., Bahceci E. (2022). Molecular profile of FLT3-mutated relapsed/refractory patients with AML in the phase 3 ADMIRAL study of gilteritinib. Blood Adv..

[33] FDA Approves Ziftomenib for Relapsed or Refractory Acute Myeloid Leukemia with a NPM1 Mutation. https://www.fda.gov/drugs/resources-information-approved-drugs/fda-approves-ziftomenib-relapsed-or-refractory-acute-myeloid-leukemia-npm1-mutation.

[34] Searle E., Recher C., Abdul-Hay M., Abedin S., Aldoss I., Pierola A.A., Alonso-Dominguez J.M., Chevallier P., Cost C., Daskalakis N. (2024). Bleximenib dose optimization and determination of RP2D from a phase 1 study in relapsed/refractory acute leukemia patients with *KMT2A* and *NPM1* alterations. Blood.

[35] Zeidner J.F., Yuda J., Watts J.M., Levis M.J., Erba H.P., Fukushima K., Shima T., Palmisiano N.D., Wang E.S., Borate U. (2024). Phase 1 Results: First-in-Human Phase 1/2 Study of the Menin-MLL Inhibitor Enzomenib (DSP-5336) in Patients with Relapsed or Refractory Acute Leukemia. Blood.

[36] Wu D., Wang Y., Chen S., Li Y., Huang R., Ren J., Guo X., Li Y., Sun M., Wei X. (2024). A First-in-Human Phase 1/2 Study of the Menin-KMT2A(MLL1) Inhibitor BN104 in Adult Patients with Relapsed or Refractory Acute Leukemia. Blood.

[37] Wenge D.V., Armstrong S.A. (2025). Menin inhibition for the treatment of acute leukemia. Semin. Hematol..

[38] Thakur R.K., Wang E.S. (2025). The promise of menin inhibitors: From approval to triplet regimens. Hematol. Am. Soc. Hematol. Educ. Program.

[39] Senapati J., Konopleva M., Issa G.C., Jabbour E., Kadia T., DiNardo C., Borthakur G., Pemmaraju N., Short N.J., Yilmaz M. (2025). A phase 1/2 study of DS-1594 menin inhibitor in relapsed/refractory acute leukemias. J. Hematol. Oncol..

[40] DiNardo C.D., Jonas B.A., Pullarkat V., Thirman M.J., Garcia J.S., Wei A.H., Konopleva M., Döhner H., Letai A., Fenaux P. (2020). Azacitidine and Venetoclax in Previously Untreated Acute Myeloid Leukemia. N. Engl. J. Med..

[41] Pratz K.W., Jonas B.A., Pullarkat V., Thirman M.J., Garcia J.S., Döhner H., Récher C., Fiedler W., Yamamoto K., Wang J. (2024). Long-term follow-up of VIALE-A: Venetoclax and azacitidine in chemotherapy-ineligible untreated acute myeloid leukemia. Am. J. Hematol..

[42] Lachowiez C.A., Loghavi S., Kadia T.M., Daver N., Borthakur G., Pemmaraju N., Naqvi K., Alvarado Y., Yilmaz M., Short N. (2020). Outcomes of older patients with NPM1-mutated AML: Current treatments and the promise of venetoclax-based regimens. Blood Adv..

[43] Yin L., Wan L., Zhang Y., Hua S., Shao X. (2024). Recent Developments and Evolving Therapeutic Strategies in *KMT2A*-Rearranged Acute Leukemia. Cancer Med..

[44] Loke J., Buka R., Craddock C. (2021). Allogeneic Stem Cell Transplantation for Acute Myeloid Leukemia: Who, When, and How?. Front. Immunol..

[45] Piccini M., Pilerci S., Merlini M., Grieco P., Scappini B., Bencini S., Peruzzi B., Caporale R., Signori L., Pancani F. (2021). Venetoclax-Based Regimens for Relapsed/Refractory Acute Myeloid Leukemia in a Real-Life Setting: A Retrospective Single-Center Experience. J. Clin. Med..

[46] Zucenka A., Pileckyte R., Trociukas I., Peceliunas V., Vaitekenaite V., Maneikis K., Davainis L., Zvirblis T., Stoskus M., Gineikiene E. (2021). Outcomes of relapsed or refractory acute myeloid leukemia patients failing venetoclax-based salvage therapies. Eur. J. Haematol..

[47] Zhou X., Zhang L., Aryal S., Veasey V., Tajik A., Restelli C., Moreira S., Zhang P., Zhang Y., Hope K.J. (2024). Epigenetic regulation of noncanonical menin targets modulates menin inhibitor response in acute myeloid leukemia. Blood.

[48] Ling Q., Zhou Y., Qian Y., Qian J., Zhang Y., Wang J., Zhu Y., Zhou Y., Wei J., Yang C. (2023). Repressing HIF-1α-induced HDAC9 contributes to the synergistic effect of venetoclax and MENIN inhibitor in *KMT2Ar* AML. Biomark. Res..

[49] Zeidner J.F., Lin T.L., Welkie R.L., Curran E., Koenig K., Stock W., Madanat Y.F., Swords R., Baer M.R., Blum W. (2025). Azacitidine, Venetoclax, and Revumenib for Newly Diagnosed *NPM1*-Mutated or *KMT2A*-Rearranged AML. J. Clin. Oncol..

[50] Issa G.C., Cuglievan B., Daver N., DiNardo C.D., Farhat A., Short N.J., McCall D., Pike A., Tan S., Kammerer B. (2024). Phase I/II Study of the All-Oral Combination of Revumenib (SNDX-5613) with Decitabine/Cedazuridine (ASTX727) and Venetoclax (SAVE) in R/R AML. Blood.

[51] Wei A.H., Reyner J.E., Garciaz S., Aldoss I., Piérola A.A., Allred A., Dominguez J.M.A., Barreyro L., Bories P., Daskalakis N. Rp2d determination of bleximenib in combination with ven+aza: Phase 1b study in ND & R/R AML with KMT2A/NPM1 alterations. Proceedings of the European Hematology Association Congress.

[52] Erba H., Wang E., Fathi A., Roboz G., Madanat Y., Strickland S., Balasubramanian S., Mangan J., Pratz K., Advani A. Ziftomenib combined with intensive induction chemotherapy (7+3) in newly diagnosed NPM1-M or KMT2A-R acute myeloid leukemia (AML): Updated phase 1a/b results from Komet-007. Proceedings of the European Hematology Association Congress.

[53] Fathi A.T., Issa G.C., Wang E.S., Erba H., Altman J.K., Balasubramanian S.K., Roboz G.J., Schiller G.J., McMahon C.M., Palmisiano N.D. (2024). Ziftomenib combined with venetoclax/azacitidine in relapsed/refractory *NPM1*-m or *KMT2A*-r acute myeloid leukemia: Interim phase 1a results from KOMET-007. Blood.

[54] Döhner H., Schuh A., Recher C., O’NIons J., Aldoss I., Alfonso-Pierola A., Allred A., Alonso-Dominguez J.M., Barreyro L., Bories P. (2025). Bleximenib in combination with intensive chemotherapy: A phase 1b study in newly diagnosed Acute Myeloid Leukemia with *KMT2A* or *NPM1* alterations. Blood.

[55] Borlenghi E., Cattaneo C., Bertoli D., Cerqui E., Archetti S., Passi A., Oberti M., Zollner T., Giupponi C., Pagani C. (2022). Prognostic Relevance of *NPM1* and FLT3 Mutations in Acute Myeloid Leukaemia, Longterm Follow-Up-A Single Center Experience. Cancers.

[56] Carter B.Z., Tao W., Mak P.Y., Ostermann L.B., Mak D., McGeehan G., Ordentlich P., Andreeff M. (2021). Menin inhibition decreases Bcl-2 and synergizes with venetoclax in *NPM1*/FLT3-mutated AML. Blood.

[57] Jiang B., Zhao Y., Luo Y., Yu J., Chen Y., Ye B., Fu H., Lai X., Liu L., Ye Y. (2024). Outcomes of Allogeneic Hematopoietic Stem Cell Transplantation in Adult Patients with Acute Myeloid Leukemia Harboring *KMT2A* Rearrangement and Its Prognostic Factors. Cell Transplant..

[58] Lacoste S.A., Gagnon V., Béliveau F., Lavallée S., Collin V., Hébert J. (2024). Unveiling the Complexity of *KMT2A* Rearrangements in Acute Myeloid Leukemias with Optical Genome Mapping. Cancers.

[59] Young A.L., Davis H.C., Challen G.A. (2023). Droplet Digital PCR for Oncogenic *KMT2A* Fusion Detection. J. Mol. Diagn..

[60] Winters A.C., Bernt K.M. (2017). *MLL*-Rearranged Leukemias-An Update on Science and Clinical Approaches. Front. Pediatr..

[61] Meyer C., Larghero P., Lopes B.A., Burmeister T., Gröger D., Sutton R., Venn N.C., Cazzaniga G., Abascal L.C., Tsaur G. (2023). The *KMT2A* recombinome of acute leukemias in 2023. Leukemia.

[62] Perner F., Stein E.M., Wenge D.V., Singh S., Kim J., Apazidis A., Rahnamoun H., Anand D., Marinaccio C., Hatton C. (2023). *MEN1* mutations mediate clinical resistance to menin inhibition. Nature.

[63] McMahon C.M. (2023). *MEN1* Mutations Induce Acquired Resistance to the Menin Inhibitor Revumenib. Hematologist.

[64] Bourgeois W., Cutler J., E Rice H., Regalado B., Wenge D.V., Perner F., Doench J.G., McGeehan J.M., A Armstrong S. (2024). Discerning the Landscape of Menin Inhibitor Resistance. Blood.

[65] Woyach J.A., Jones D., Jurczak W., Robak T., Illés Á., Kater A.P., Ghia P., Byrd J.C., Seymour J.F., Long S. (2024). The menin-*KMT2A* inhibitor JNJ-75276617 targets leukemia cells harboring *MEN1* resistance mutations. Blood.

[66] Perner F., Rahnamoun H., Wenge D.V., Xiong Y., Apazidis A., Anand D., Hatton C., Wen Y., Gu S., Liu X.S. (2023). Non-Genetic Resistance to Menin Inhibition in AML Is Reversible by Perturbation of KAT6A. HemaSphere.

[67] Zheng H., Xian H., Zhang W., Lu C., Pan R., Liu H., Xu Z. (2025). MENIN inhibitor-based therapy in acute leukemia: Latest updates from the 2024 ASH annual meeting. J. Hematol. Oncol..

[68] Candoni A., Coppola G. (2024). A 2024 Update on Menin Inhibitors. A New Class of Target Agents against KMT2A-Rearranged and NPM1-Mutated Acute Myeloid Leukemia. Hematol. Rep..

[69] Issa G.C., Aldoss I., Thirman M.J., DiPersio J., Arellano M., Blachly J.S., Mannis G.N., Perl A., Dickens D.S., McMahon C.M. (2024). Menin Inhibition with Revumenib for *KMT2A*-Rearranged Relapsed or Refractory Acute Leukemia (AUGMENT-101). J. Clin. Oncol..

[70] Stutterheim J., Van der Sluis I.M., Vrenken K.S., Pieters R., Stam R.W. (2025). *KMT2A*-rearranged acute lymphoblastic leukemia in infants: Current progress and challenges. Haematologica.

[71] Forghieri F., Nasillo V., Paolini A., Bettelli F., Pioli V., Giusti D., Gilioli A., Colasante C., Acquaviva G., Riva G. (2020). *NPM1*-Mutated Myeloid Neoplasms with <20% Blasts: A Really Distinct Clinico-Pathologic Entity?. Int. J. Mol. Sci..

[72] Matthews A.H., Pratz K.W., Carroll M.P. (2022). Targeting Menin and CD47 to Address Unmet Needs in Acute Myeloid Leukemia. Cancers.

[73] Huang C.Y., Huang W.K., Yeh K.Y., Chang J.W., Lin Y.C., Chou W.C. (2025). Integrating comprehensive genomic profiling in the management of oncology patients: Applications and challenges in Taiwan. Biomed. J..

[74] Syndax Pharmaceuticals (2025). Syndax’s Revuforj (revumenib) included in NCCN Clinical Practice Guidelines in Oncology (NCCN Guidelines) for the treatment of relapsed or refractory *NPM1* mutated acute myeloid leukemia. https://ir.syndax.com/news-releases/news-release-details/syndaxs-revuforjr-revumenib-included-nccn-clinical-practice.

